# Comparative Efficacy of Antihypertensive Agents in Flow-Mediated Vasodilation of Patients with Hypertension: Network Meta-Analysis of Randomized Controlled Trial

**DOI:** 10.1155/2022/2432567

**Published:** 2022-04-30

**Authors:** Hong Ding, Shu Liu, Ke-Xin Zhao, Jie Pu, Ya-Fei Xie, Xiao-Wei Zhang

**Affiliations:** Department of Cardiology, Lanzhou University Second Hospital, Lanzhou, China

## Abstract

Hypertension induces both structural and functional changes in blood vessels, thereby increasing endothelial dysfunction, which in turn, contributes to an increase in blood pressure. A popular and widely used noninvasive tool, flow-mediated dilation (FMD), is used to examine peripheral artery endothelium-dependent dilation. This study aimed to compare the efficacies of different classes of antihypertensive agents based on their effects on FMD. PubMed, Embase, and Cochrane Library were queried till November 1, 2020. Comparative studies on the efficacies of two or more antihypertensive agents or placebos for hypertensive patients were included. The outcomes were variations in mean systolic and diastolic blood pressure. Two reviewers independently reviewed and filtered the literature and extracted the data; the Cochrane “risk of bias” method was used to evaluate the methodological quality of the randomized controlled trials. A network meta-analysis was performed using Stata 15.0 software with a total of 49 studies. Subgroup analysis based on age and duration of treatments was performed. As compared to the placebo group, patients receiving the antihypertensive drugs exhibited significantly enhanced FMD (ARB + CCB: 4.01%, 95% CI, 0.92–7.11%, *p* < 0.001; ACEI + ARB: 2.81%, 95% CI, 1.19–4.43%, *p* < 0.001; ACEI: 2.55%, 95% CI, 1.34–3.77%, *p* < 0.001; ARB: 2.22%, 95% CI, 1.05–3.38%, *p* < 0.001; *β*-blocker: 2.23%, 95% CI, 0.93–3.52%, *p* < 0.001). In the SUCRA curve for network meta-analysis, the combination of CCB and ARB was found to be the most effective in increasing FMD (SUCRA = 89.0%), followed by ACEI monotherapy (SUCRA = 74.2%). ARB combined with CCB was superior in improving the endothelial function measured as the FMD; ACEI monotherapy was the most effective treatment among the antihypertension medications. There were no significant differences between antihypertensive drug-based monotherapies.

## 1. Introduction

Hypertension contributes significantly to the total disease burden and is an important cause of mortality. A CHS study (2012–2015) reports that the prevalence of hypertension among Chinese residents (≥18 years old) is 27.9% [1]. Elevated blood pressure (BP) has an intimate and continuous association with cardiovascular (CV) and renal events [2]. Estimates suggest that, in 2025, approximately 1.5 billion adults would be hypertensive [2].Based on epidemiological survey, the prevalence of hypertension would be over 150 million in central and Eastern Europe [2]. Hypertension induces structural and functional changes in blood vessels that increase endothelial dysfunction and subsequently may also contribute to an increase in the blood pressure [3].Thus, the possibility of ameliorating the impaired endothelial function is an important target for the antihypertensive therapy [4]. Flow-mediated dilation (FMD) is the most frequently noninvasive technique for the evaluation of endothelial function by brachial ultrasound during reactive hyperemia [5].

Knowledge of the optimal antihypertension medications for the prevention of FMD will be crucial for decision-making in clinical settings. Moreover, the identification of the most effective treatments for controlling hypertension and management of impaired endothelial function is imperative to guide clinicians and decrease the global burdens of cardiovascular diseases.

Previous meta-analyses have examined the efficacy of antihypertension treatments for improving the FMD status using [6, 7] pairwise comparisons of only two classes of antihypertension medications; however, pairwise meta-analysis does not enable comparisons among multiple classes of medications. To provide an updated perspective on the comparative efficacies of antihypertension medications, we performed a network meta-analysis to compare the reported effects of different classes of antihypertension medications that are in current use for increasing FMD. FMD is strictly dependent on the brachial artery diameter. Therefore, we also performed a network meta-analysis to examine the drug-induced changes on comparisons of the brachial artery diameter (BAD) in the literature.

## 2. Materials and Methods

### 2.1. Search Strategy

Electronic databases including PubMed, Cochrane Library, Embase, and ClinicalTrials.gov were systematically queried for English versions of the publications until November 1, 2020. PICO criteria were as follows: (1) population: hypertensive adults with the mean age of 18 years or above; (2) intervention and comparison: at least two different classes of antihypertension medications or one antihypertension medication intervention with placebo controlled intervention compared in each trial; (3) outcome: flow-mediated dilation and brachial artery diameter. We used a combination of Medical Subject Headings (MeSH) terms and words to retrieve the relevant articles on directive comparisons on the efficacies of different classes of antihypertensive medications in FMD of hypertensive patients. The search terms used were as follows: hypertension AND flow-mediated dilation AND antihypertension medications (refer to Appendix). All the bibliographies of the selected articles were screened to collect the additional relevant articles. When the full-text articles were not accessible through the electronic databases, we contacted corresponding authors by phone or through e-mail. Gray literature has been retrieved, but the literature is not related, and not included.

### 2.2. Study Inclusion Criteria

The titles and abstracts were construed by two independent reviewers and evaluated for the inclusion and exclusion criteria through EndNote software and duplicates were removed automatically. The third reviewer was consulted in case of discrepancies regarding the study selection, which was then resolved. Prespecific selection criteria include the following: (1) the studies were prospective, randomized, controlled trials; (2) patients ＞18 years; (3) adult patients satisfying the diagnosis criteria of hypertension (SBP ≥140 mmHg or DBP ≥90 mmHg) or previously diagnosed with hypertension; (4) random assignment to different classes of antihypertensive agents, and (5) the studies reporting the available index of FMD. After assessment of the full text, the articles that met the selection criteria and provided sufficient data were included for further analysis.

### 2.3. Data Extraction and Quality Assessment

According to the predefined selection criteria, data were extracted independently by the two reviewers. The articles with not available outcome data were excluded. Any inconsistencies were resolved by discussion, and when necessary, cross-validated with the authors of the trial study. The following data were extracted from each trial: author, country, sample size, age, BMI, sex, antihypertensive medication, dose, time, blood pressure, FMD methodology, and outcomes measured. The methodological quality of each study was evaluated by the two reviewers according to guidelines in chapter 8 of the Cochrane handbook [8–10].

### 2.4. Statistical Analysis

The network meta-analysis in our study was performed using a Bayesian random-effects generalized linear model. The results were reported as the standard mean deviation of 95% confidence intervals (CI) after the comparison of all intervention modes. The hypothesis test was a *U*-test; the value of *P* < 0.05 was considered statistically significant. In a closed-loop, the inconsistencies between direct and indirect evidence were evaluated by the node splitting method. The efficacy of the intervention was ranked based on the surface values under the cumulative ranking (SUCRA) curve [11]. Finally, a funnel plot was generated to detect potential the publication bias. The sensitivity was analyzed according to random effects model. We performed a subgroup analysis based on the age and duration of treatments. The mean age of participants was used to classify the studies into two groups, namely, trials that had enrolled patients aged ≤55 years and trials with patients aged ＞55 years. Based on the treatment duration, the studies were categorized based on antihypertension medications. All statistical analyses were performed using Review Manager 5.3 (The Cochrane Collaboration) and Stata 15.1.

## 3. Results

### 3.1. Characteristics of the Included Studies

**Figure 1:**
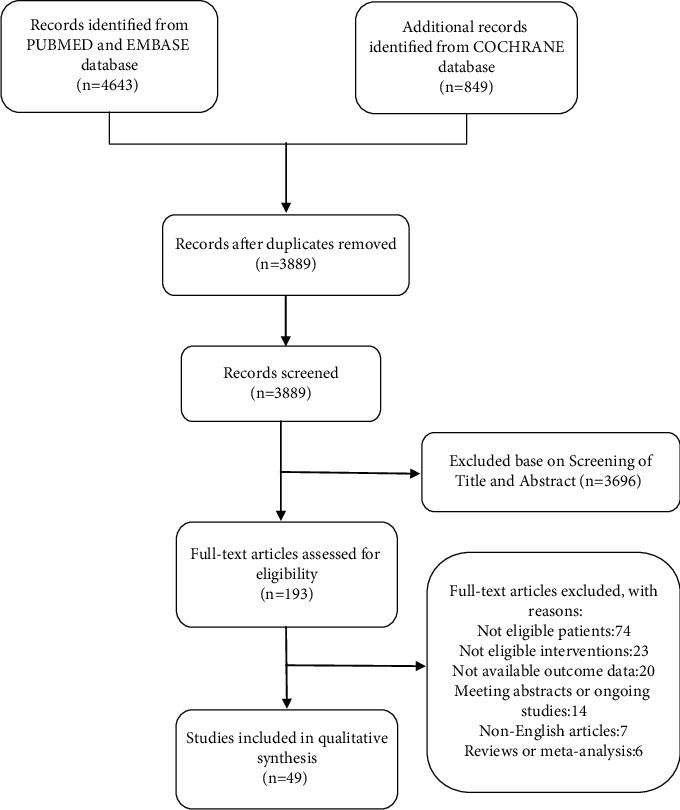
PRISMA diagram of the clinical review search strategy.

49 randomized controlled trials (RCTs) comprising 2646 patients suffering from antihypertension and treated with corresponding antihypertensive agents for improving their FMD status were included to determine the contributions of these agents in enhancing FMD. The studies included in our meta-analysis were all RCTs, published until November 2020. The studies were conducted in Italy [12–19], Germany [20], China [21–26], Turkey [27–33], UK [34], Brazil [35–37], Greece [38], Denmark [39], Korea [40–45], Bulgaria [46], USA [47], Chile [48], and Japan [49–60]. A flow diagram depicting the inclusion process of these studies is shown in Figure 1.  Table 1 presents the basic characteristics of the included trials and the demographic data of the participants.

### 3.2. Quality of the Included Studies

**Figure 2:**
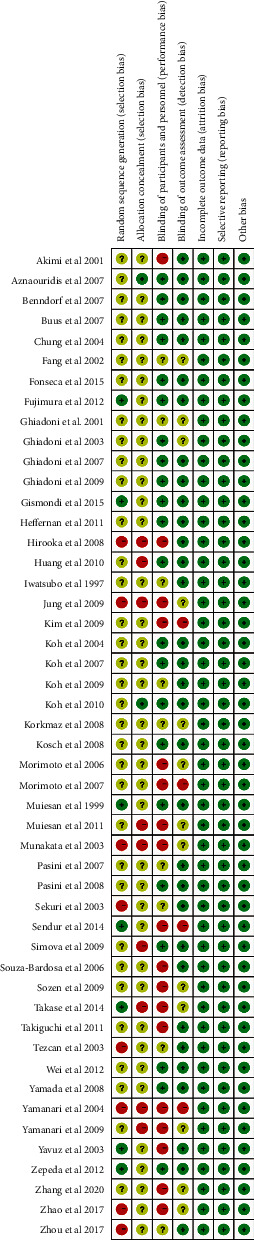
Methodological quality summary: review authors' judgments about each methodological quality.

**Figure 3:**
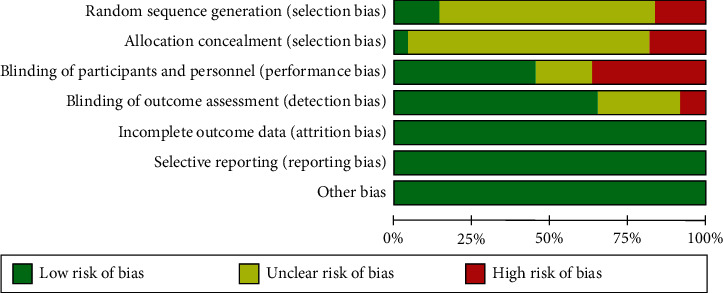
Methodological quality graph: reviewer author's judgments about each methodological quality item presented as percentage across all included studies.

The quality of all included RCTs was assessed using the tools of the Cochrane Collaboration. Randomization was performed in all RCTs. However, only six studies described the method of randomization, which included sealed envelopes, random number table, and randomly permuted blocks. RCTs included in this study provided complete data and but not provide information on other potential biases. The qualities of the article evaluated are as follows (Figures 2 and 3).

### 3.3. Network Geometry

**Figure 4:**
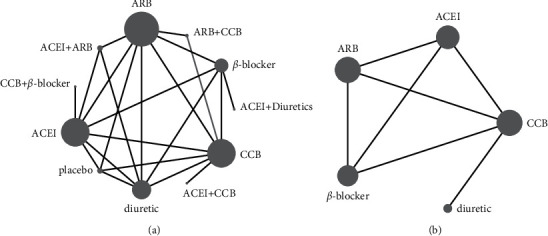
The construction of the network. (a) Flow-mediated dilation, (b) brachial artery diameter) ACEI: angiotensin-converting enzyme inhibitors, ARB: angiotensin receptor blocker, CCB: calcium channel blockers.

As shown in the network diagram, each point represents a drug, and a directly connected line segment between the two points, indicated that a direct comparison between the two drugs was reported. The size of the nodes and the width of the lines are directly proportional to the number of tests. Network evidence for the comparisons between the different antihypertensive agents is shown in Figure 4.

### 3.4. Testing for Inconsistency

**Figure 5:**
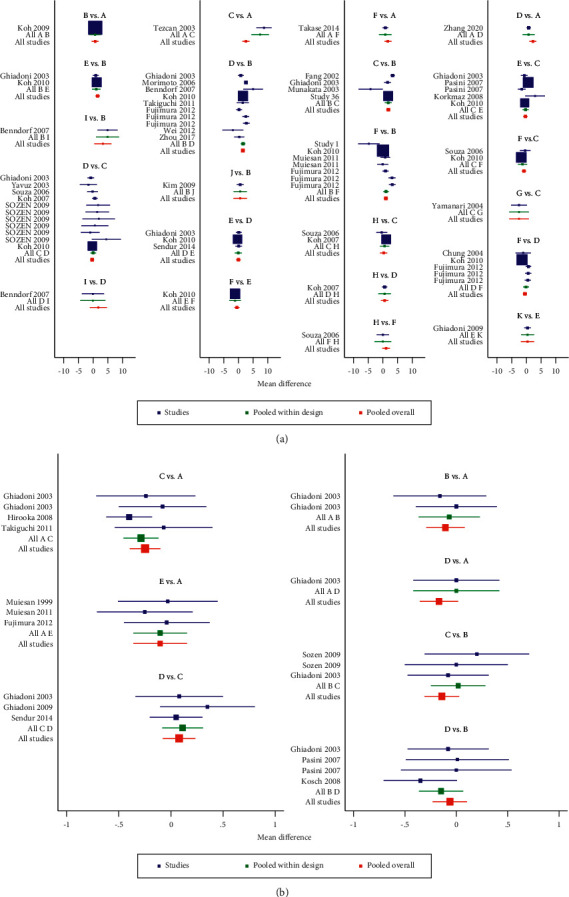
Results of test for inconsistency. ((a) flow-mediated dilation, (b) brachial artery diameter). A, placebo; B, CCB (calcium channel blockers); C, ACEI (angiotensin-converting enzyme inhibitors); D, ARB (angiotensin receptor blocker); E, *β*-blocker; F, diuretic; G, CCB + *β*-blocker; H, ACEI + ARB; I, ARB + CCB; G, ACEI + CCB.

**Figure 6:**
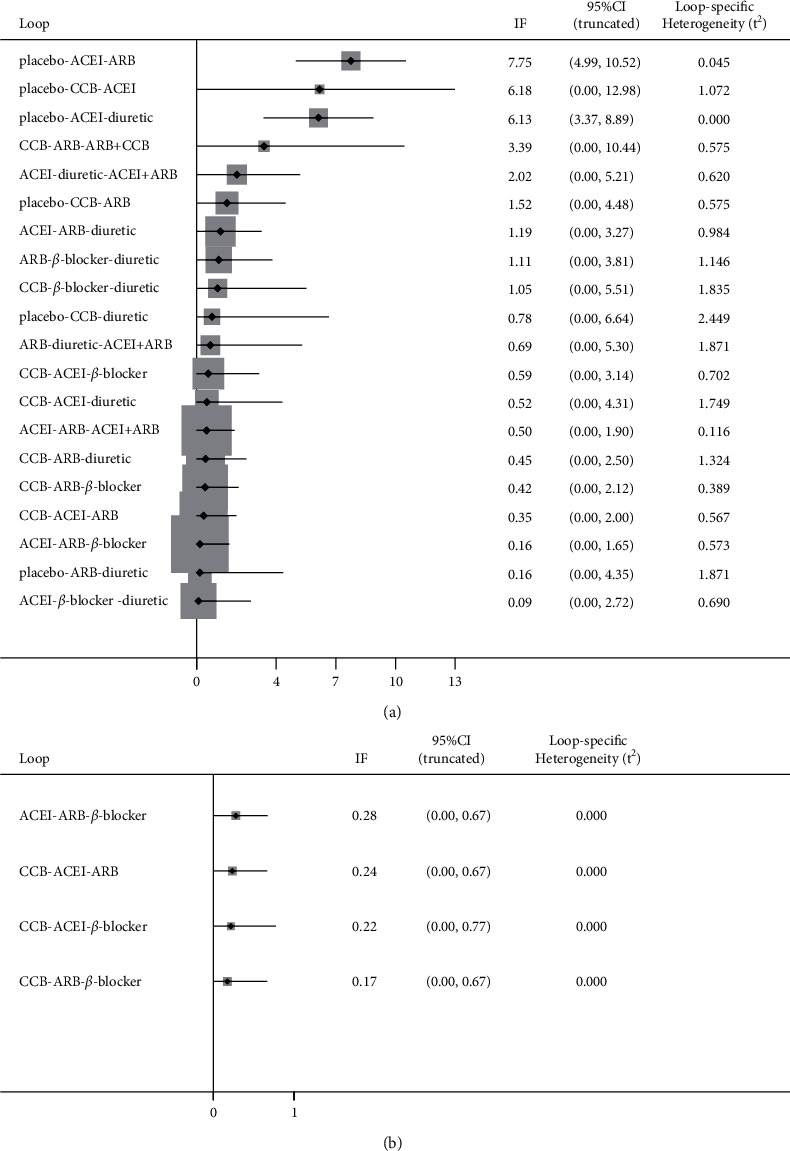
Results of test for inconsistency. (a) Flow-mediated dilation, (b) brachial artery diameter) ACEI: angiotensin-converting enzyme inhibitors, ARB: angiotensin receptor blocker, CCB: calcium channel blockers.

The inconsistency test showed that the comparison could be valued for consistency, *χ*^2^ = 17.35, *P*=0.1368 in FMD (Figures 5(a) and 6(a)) and *χ*^2^ = 3.01, *P*=0.390 in BAD (Figures 5(b) and 6(b)). Based on the *P*-values, the results of inconsistency tests between direct and indirect treatment comparisons for mixed treatment comparison showed no general inconsistencies between treatment effects on each outcome (all *P* > 0.05, Tables 2 and 3).

### 3.5. Effect of Antihypertensive Medications on FMD and BAD

**Figure 7:**
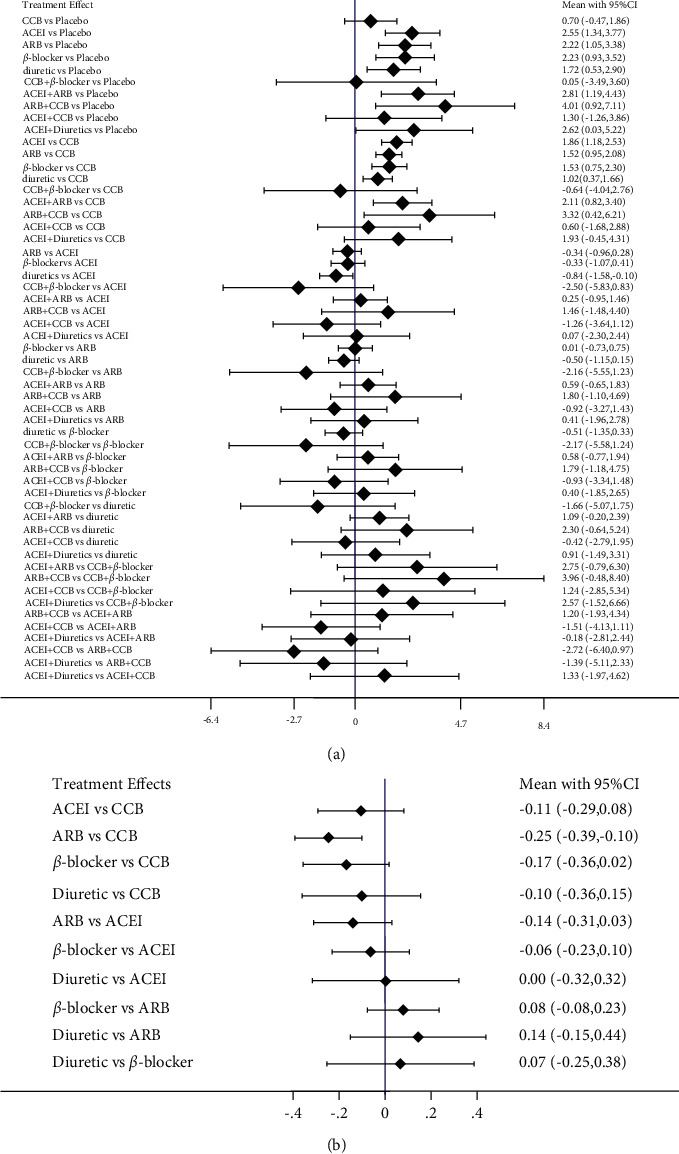
Absolute mean (95% credible interval, CRI) difference. (a) Flow-mediated dilation, (b) brachial artery diameter). ACEI: angiotensin-converting enzyme inhibitors, ARB: angiotensin receptor blocker, CCB: calcium channel blockers.

**Figure 8:**
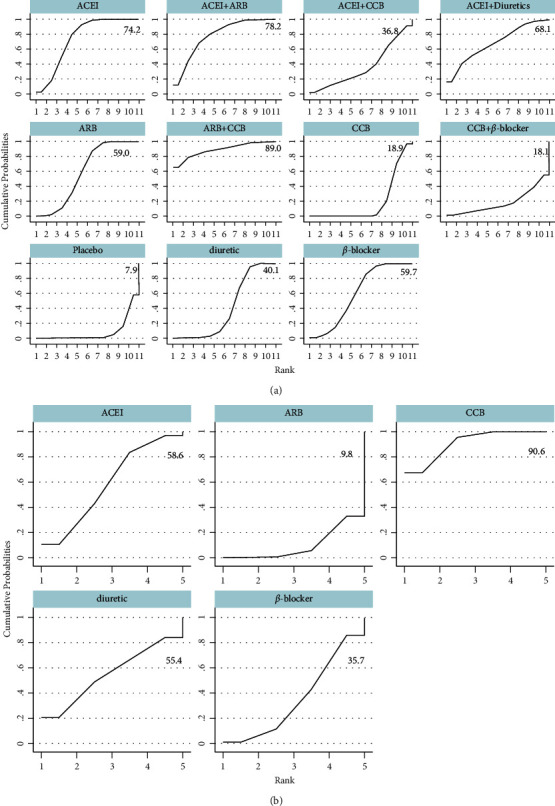
Mean ranking plots. (a) Flow-mediated dilation, (b) brachial artery diameter). ACEI: angiotensin-converting enzyme inhibitors, ARB: angiotensin receptor blocker, CCB: calcium channel blockers.

Compared with the placebo group, the antihypertensive drugs significantly enhance FMD [angiotensin receptor blocker (ARB)] + calcium channel blockers (CCB): 4.01%, 95% CI, 0.92–7.11%, *p* < 0.001; angiotensin-converting enzyme inhibitors (ACEI) + ARB: 2.81%, 95% CI, 1.19–4.43%, *p* < 0.001; ACEI: 2.55%, 95% CI, 1.34–3.77%, *p* < 0.001;ARB: 2.22%, 95% CI, 1.05–3.38%, *p* < 0.001; *β*-blocker: 2.23%, 95% CI, 0.93–3.52%, *p* < 0.001). No significant differences between monotherapy (Figure 7(a)). In the SUCRA curve for network meta-analysis in FMD, the combination of CCB and ARB was found to be the most effective agent for increasing FMD (SUCRA = 89.0%), followed by ACEI mono-therapy (SUCRA = 74.2%) (Figure 8(a)). In BAD, no statistically significant differences among antihypertension medications were observed (Figure 7(b)). The SUCRA curve for network meta-analysis demonstrated that the most effective antihypertension medication was CCB; it could increase BAD substantially (SUCRA = 90.6%, Figure 8(b)).

### 3.6. Subgroup Analysis for FMD

**Figure 9:**
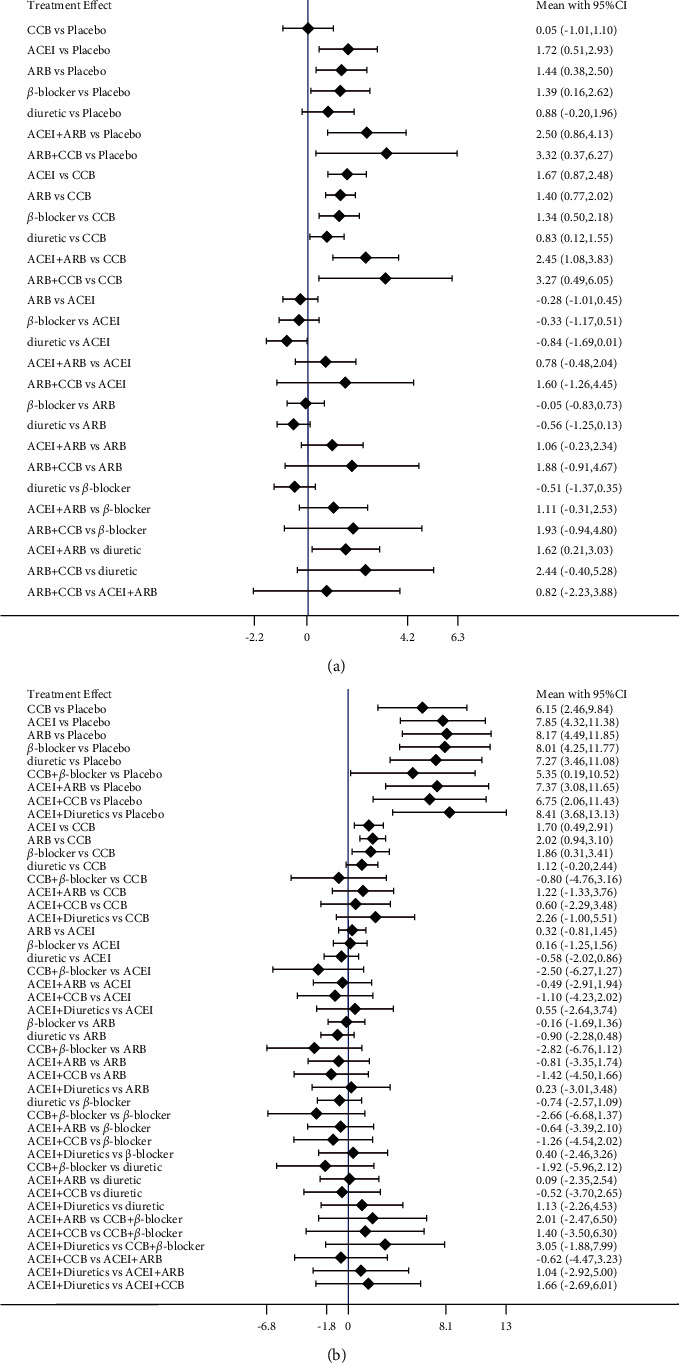
Subgroup analysis of forest plots for the assessment of duration treatments in FMD. (a) Duration treatments ≤8 weeks, (b) duration treatments ＞8 weeks). ACEI: angiotensin-converting enzyme inhibitors, ARB: angiotensin receptor blocker, CCB: calcium channel blockers.

**Figure 10:**
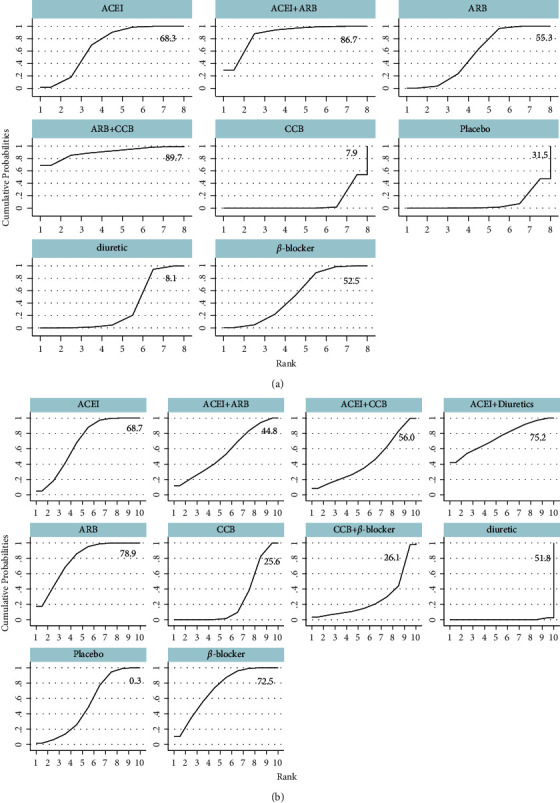
Subgroup analysis of cumulative ranking probability plot for the assessment of duration treatments in FMD. (a) Duration treatments ≤8 weeks, (b) duration treatments ＞8 weeks) ACEI: angiotensin-converting enzyme inhibitors, ARB: angiotensin receptor blocker, CCB: calcium channel blockers.

**Figure 11:**
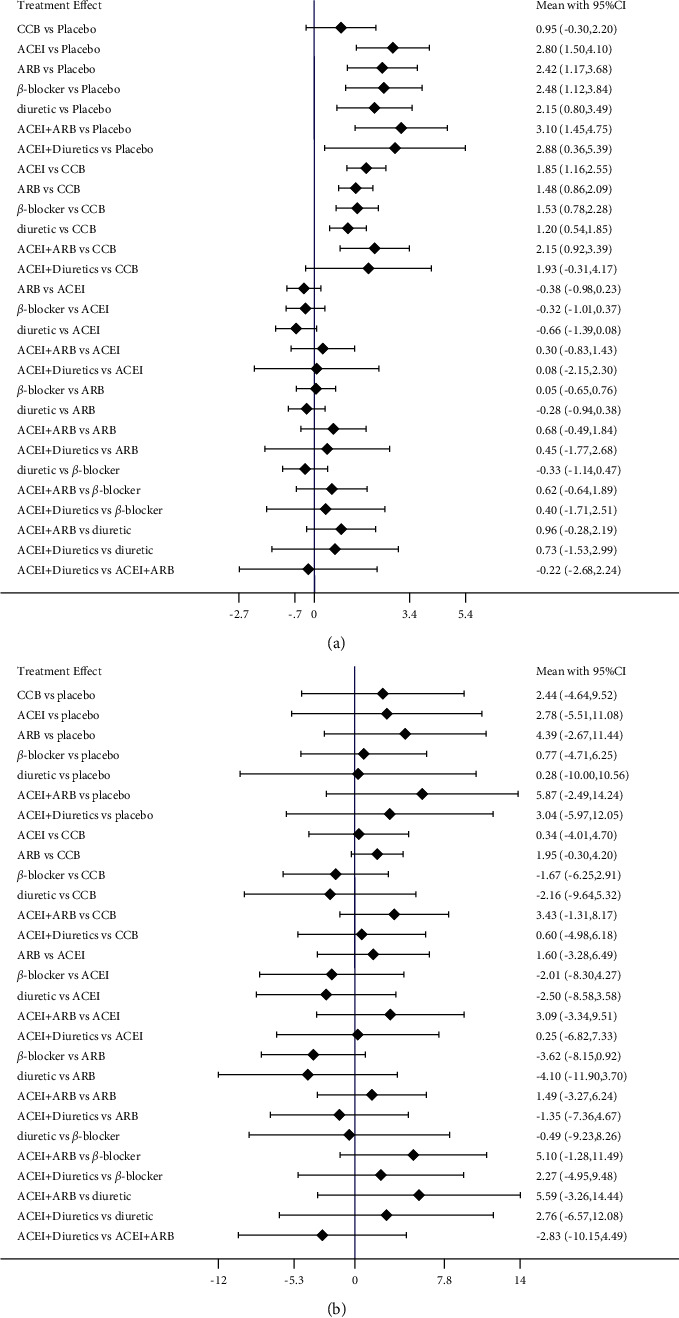
Subgroup analysis of forest plots for the assessment of age in FMD. (a) Age ≤55 years, (b) aged ＞55 years). ACEI: angiotensinconverting enzyme inhibitors, ARB: angiotensin receptor blocker, CCB: calcium channel blockers.

**Figure 12:**
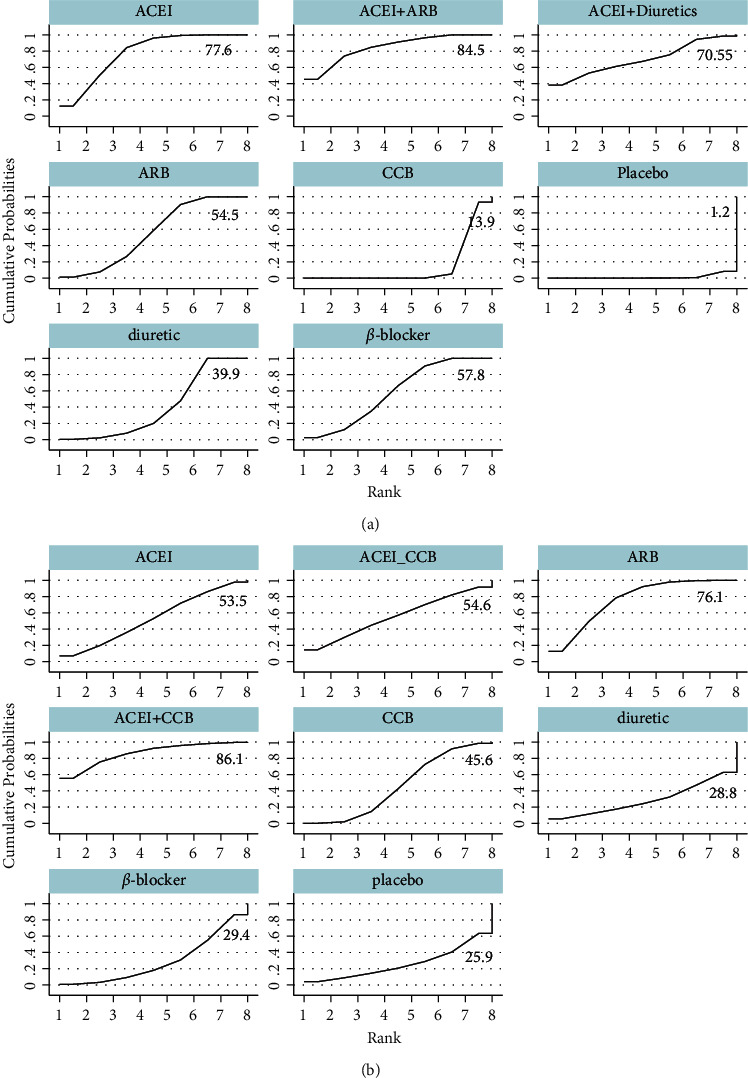
Subgroup analysis of cumulative ranking probability plot for the assessment of duration treatments in FMD. (a) Age ≤55 years, (b) aged ＞55 years). ACEI: angiotensin-converting enzyme inhibitors, ARB: angiotensin receptor blocker, CCB: calcium channel blockers.

In the subgroup analysis for FMD, the combination of CCB and ARB followed by ACEI mono-therapy (SUCRA = 68.3%), exerted beneficial effects in increasing FMD for treatment duration ≤8 weeks (SUCRA = 89.7%). For treatment duration ＞8 weeks, such significant differences were absent for combination of CCB and ARB treatment among the available studies. ARB mono-therapy had superior effects as compared to other antihypertensive drugs (SUCRA = 78.9%) (Figures 9 and 10). For patients aged ≤55 years, no significant differences due to the combination of CCB and ARB were observed. ACEI was significantly associated with an increase in FMD in comparison with other antihypertensive drugs (SUCRA = 78.9%). When compared with other antihypertensive drugs, the combination of CCB and ARB was associated with a significant increment in FMD in the participants aged ＞55 years included (SUCRA = 86.1%) (Figures 11 and 12).

### 3.7. Publication Bias

**Figure 13:**
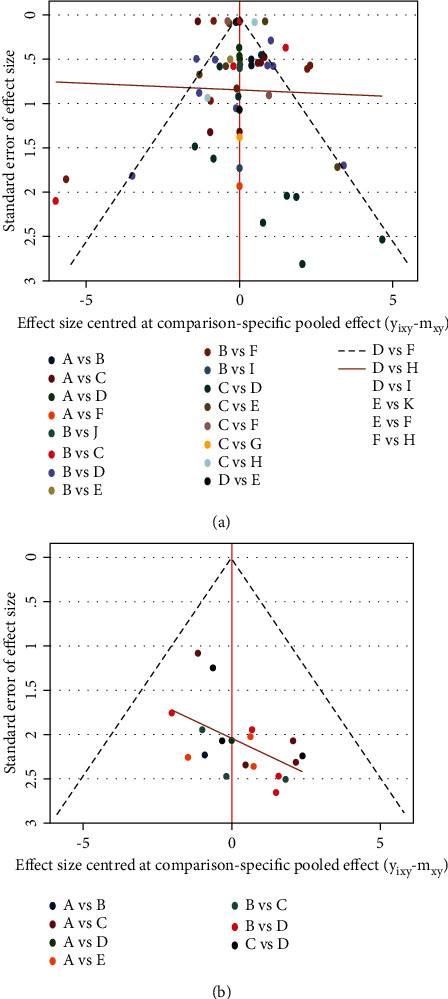
Funnel plot. ((a) flow-mediated dilation, (b) brachial artery diameter) A, placebo; B, CCB (calcium channel blockers); C, ACEI (angiotensin‐converting enzyme inhibitors); D, ARB (angiotensin receptor blocker); E, *β*-blocker; F, diuretic; G, CCB + *β*-blocker; H, ACEI + ARB; I, ARB + CCB; G, ACEI + CCB.

Potential publication biases in reporting effects of antihypertensive agents for the treatment of patients with hypertension were evaluated and shown as a funnel plot (Figure 13); not all studies were symmetrically distributed around the vertical line, *X* = 0, which indicated that there was evidence of small sample effect in the research network.

### 3.8. Sensitivity Analysis

Sensitivity analysis was performed according to random effects model to evaluate the stability and reliability of the combined results of the meta-analyses and assess whether the combined results were unduly affected by the results of a single study. This procedure was conducted using Stata. After eliminating the individual studies one by one, most of the combined effect sizes were relatively minor, which indicated that the results of this meta-analysis were relatively stable.

## 4. Discussion

A total of 49 RCTs examining the effects of the most commonly prescribed antihypertension medications for improving endothelial function as measured by brachial FMD, were included in the present analysis. The pooled results showed that a combination of CCB and ARB, followed by ACEI (SUCRA = 74.2%) exerted synergistic effects in protecting the endothelial function (SUCRA = 89.0%) by enhancing the FMD. The network meta-analysis also showed that CCB played a better role in improving the BAD value (SUCRA = 90.6%). Our study provided the most current evidence on the comparative efficacy of antihypertensive medications, which have been previously in improving vascular function in RCTs. Furthermore, to the best of our knowledge, this is the first network meta-analysis that pools the results of studies that have tested the efficacies of antihypertension medications on protecting endothelial functions by increasing FMD.

FMD has been widely used for the assessment of endothelial function in humans owing to its non-invasive nature [61]. A recent meta-analysis concludes that a significantly lower risk (8–13%) of CV events per percentage point increase in brachial artery FMD occurs [62]. Our network meta-analysis showed that the combination of CCB and ARB significantly increased FMD by 4.01% and 3.32% as compared to placebo and CCB, respectively. Results in this network meta-analysis are consistent with those reported in previous meta-analyses of RCTs. Miroslav et al [63] found that CCB was efficient and effective for the improvement of the FMD parameters. Michał et al [64] indicated that beta-blockers can significantly improve the endothelial function as compared to placebo. However, theses meta-analyses found no statistical differences among the efficacies of the antihypertensive medications. Yousef et al [6] report that ACEI mono-therapy is the most effective treatment regime for improving FMD as compared to CCB and beta-blockers. Jian-Dong Chen et al. [7] also found no significant difference among the different classes of antihypertensive drugs on FMD. Our findings further support the evidence for ACEI monotherapy among the antihypertensive medications, as the most effective treatment for improving FMD. Our finding was in contrast with a previous study, and indicated that a combination of CCB and ARB was the most effective treatment for improving FMD. Mechanistic insights into the effects of antihypertensive medications on FMD have not been fully elucidated, however plausible explanations have been proposed. Oxidative stress [65] and inflammation [66] are the main causes of hypertension-related endothelial dysfunction, as both significant reduce the bioavailability of nitric oxide [67].Beyond this, elevated blood pressure may damage endothelial cells and, cause their irreversible damage [68]. ARB can promote the release of nitric oxide and accelerate the effect of acetylcholine on endothelium-dependent vasodilation [69]. These compounds can reverse endothelial dysfunction spontaneously hypertensive rats [70] and ameliorate FMD in patients with hypertension. CCB not only effectively reduces blood pressure but also increases the production of endothelial nitric oxide synthase, thereby improving nitric oxide bioavailability and endothelial function [7]. The results of some clinical trials show that CCB is frequently combined with ARB and improves endothelial dysfunction, thus it can be reasonably concluded that the improvement of endothelial function is significantly dependent upon the synergistic or additive pleiotropic actions of the aforementioned drugs. ARB combined with CCB is recommended in many guidelines for slowing down the progression of hypertension [71]. In experimental conditions, endothelium-dependent vasodilation was shown to benefit from the synergistic effect and complementary mechanism of the combination of CCB and ARB causes the amelioration of both oxidative stress and the impaired Akt/eNOS pathway, along with inhibition of ERK activation [72]. Evidence from clinical and experimental data also supports the use of ARB combination with CCB, which may provide superior vascular protection through an enhanced anti-inflammatory mechanism [25]. Simultaneously, the network meta-analysis also indicated the beneficial effects of the combination of ACEI and ARB on FMD (SUCRA = 78.2). However, the combined effect of the two drugs on FMD was not statistically significant as compared to the effect of either of these drugs individually. ACEI could also dramatically enhance FMD (SUCRA = 74.2) by inhibiting the angiotensin-converting enzyme, thereby suppressing the angiotensin II activity and increasing bradykinin production. Baseline BAD is an important determinant of FMD of the brachial artery, where in the FMD is calculated as a relative percentage change in the baseline BAD during reactive hyperemia. CCB lowers blood pressure by inhibiting the L-type calcium channel involved in the influx of calcium ions, leading to vascular smooth muscle relaxation and consequent peripheral vasodilation [73].

Taken together, ARB combined with CCB may be more beneficial for alleviating endothelial dysfunction in patients with hypertension. As hypertension-associated mechanisms differ among the patients, the effectiveness of antihypertension medications varies among individual patients, thereby offering reasonable effects of clinical therapy. The purpose of this network meta-analysis was to identify the most effective antihypertensive drugs for increasing FMD in patients suffering from hypertension. The advantage of network meta-analysis lies in that, the indirect comparisons among various drugs used in clinical practice, exerting the same efficacies based on pairwise head-to-head direct randomized tests and ranking in order of the treatments to identify the superior ones among them, which helps in optimizing the therapeutic strategies. Therefore, it overcomes the shortcomings of conventional meta-analysis based on pairwise head-to-head direct comparison. However, this study also has some limitations. First, the network meta-analysis in this study may be limited by the selective reporting biases and small sample sizes. Second, the findings should be considered with caution, owing to the possibility of overestimation of the therapeutic effect, as the negative results have not been published. Third, only the articles published in English were included in this network meta-analysis, which may cause selection bias. Finally, a possibility of inaccuracy in the information provided in published articles and online clinical research reports exists. In addition, most of the studies included were single-center studies without any specific description of randomization and blinding, which might bias the accuracy of the findings to uncertain extent. In order to increase the power of this meta-analysis, both RCTs were included in this network meta-analysis. Therefore, it is necessary to design rigorous large-sample, multicenter, RCTs to further study the effectiveness of antihypertensive drugs on FMD in patients with hypertension, and include experimental data to support the characteristics of various therapeutic drugs.

In conclusion, the meta-analysis presented here indicated that ARB combined with CCB was superior in improving the endothelial function measured as FMD status. ACEI mono-therapy was the most effective treatment for increasing FMD among all the antihypertension medications. There was no significant difference between mono-therapeutic antihypertensive drugs.

## Figures and Tables

**Table 1:** Characteristics of the studies included in this Meta-analysis.

Author, year	Country	Antihypertensive drug	Dose	*n*	Time	Age (year)	BMI	Sex (M/F)	Durations (year)	SBP (mmHg)	DBP (mmHg)	Measurement
Iwatsubo et al., 1997 [50]	Japan	Temocapril	2 or 4 mg	15	6 m	63 ± 3	21.1 ± 0.5	4/11	NA	160 ± 4	165 ± 5	Vascular ultrasonography
		Amlodipine	2.5 or 5 mg	11	6 m	61 ± 2	22.9 ± 0.5	3/8	NA	94 ± 2	94 ± 3	
Muiesan et al., 1999 [12]	Italy	Nifedipine	NA	10	2 m	56 ± 2	NA	7/3	NA	161 ± 16	102 ± 9	Vascular ultrasonography
		HCTZ	NA	10	2 m	56 ± 7	NA	7/3	NA	154 ± 11	98 ± 4	
Akimi, 2001	Japan	Quinapril	20 mg	15	3 m	60 ± 11	NA	9/6	NA	165 ± 16	98 ± 6	Vascular ultrasonography
		Nitrendipine	10 mg	11	3 m	58 ± 12	NA	4/7	NA	160 ± 15	92 ± 5	
Ghiadoni et al., 2001 [13]	Italy	Nifedipine	20 mg	32	2 h	52.1 ± 10.1	NA	20/12	NA	152.7 ± 10.7	101.7 ± 8.8	Vascular ultrasonography
		Captopril	50 mg	32	2 h	50.9 ± 9.6	NA	20/12	NA	150.5 ± 10.3	100.5 ± 8.8	
Fang et al., 2002 [21]	China	Nifedipine	5 mg	47	3 m	57 ± 8	NA	25/22	NA	164 ± 16	96 ± 8	Vascular ultrasonography
		Ramipril	10 mg	49	3 m	56 ± 6	NA	27/22	NA	162 ± 14	98 ± 9	
Ghiadoni et al., 2003 [14]	Italy	Nifedipine	30–60 mg	28	6 m	52 ± 11	NA	17/11	NA	153 ± 8	102 ± 2	Vascular ultrasonography
		Amlodipine	5–10 mg	28	6 m	53 ± 8	NA	17/11	NA	152 ± 9	98 ± 9	
		Atenolol	5–100 mg	29	6 m	53 ± 9	NA	18/11	NA	156 ± 10	99 ± 8	
		Nebivolol	5–10 mg	28	6 m	53 ± 8	NA	17/11	NA	152 ± 9	98 ± 9	
		Telmisartan	80–160 mg	29	6 m	50 ± 9	NA	18/11	NA	151 ± 10	100 ± 7	
		Perindopril	2–4 mg	28	6 m	51 ± 11	NA	18/10	NA	153 ± 9	100 ± 6	
Munakata et al., 2003	Japan	Amlodipine	2.5 mg	10	12 m	55 ± 2	NA	8/16	NA	172 ± 4	101 ± 3	Vascular Ultrasonography
		Benidipine	4 mg	9	12 m							
		Nifedipine	10 mg	5	12 m							
		Temocapril	2 mg	9	12 m	57 ± 3	NA	7/5	NA	172 ± 5	103 ± 2	
		Cirazapril	0.5 mg	3	12 m							
Sekuri et al., 2003 [27]	Turkish	Perindopril + indapamide	2 mg + 0.625 mg	29	6 m	54.5 ± 9.5	NA	12/17	NA	155.2 ± 10.3	96.6 ± 6.1	Vascular ultrasonography
Tezcan et al., 2003 [28]	Turkey	Enalapril	5–40 mg	9	6 m	38.3 ± 9	25.2 + 4.0	4/5	2.3 ± 2.2	147.4 ± 10.1	96.6 ± 7.0	Vascular ultrasonography
Yavuz et al., 2003 [29]	Turkey	Enalapril	5–40 mg	12	6 m	38.6 ± 7.9	24.7 ± 4.9	4/8	3.1 ± 3.7	149 ± 11	98 ± 7	Vascular ultrasonography
		Losartan	50–100 mg	12	6 m	42.2 ± 12.8	24.4 ± 4.5	4/7	3.3 ± 3	150 ± 21	100 ± 5	
Chung et al., 2004 [34]	UK	Losartan	50–100 mg	21	12 w	55.9 ± 11.8	28.3 ± 4.7	28/12	NA	164 ± 15	97 ± 11	Vascular ultrasonography
		HCTZ	12.5–25 mg	19	12 w					165 ± 18	93 ± 13	
Koh et al., 2004 [45]		Losartan	100 mg	47	2 m					147 ± 3	91 ± 1	Vascular ultrasonography
		Placebo	NA		2 m					145 ± 2	90 ± 1	
Yamanari et al., 2004 [49]	Japan	Enalapril	10 mg	10	6 m	65 ± 10		5/5	9 ± 6	NA	NA	Vascular Ultrasonography
		Manidipine + metoprolol	20 + 60 mg	11	6 m	66 ± 9		7/4	8 ± 5	NA	NA	
Morimoto et al., 2006 [53]	Japan	Amlodipine	5 mg	22	24 w	58 ± 2	23.9 ± 0.7	9/13	5 ± 1	163 ± 3	93 ± 1	Vascular ultrasonography
		Telmisartan	40 mg	21	24 w	56 ± 2	24.7 ± 0.8	9/12	7 ± 1	162 ± 5	95 ± 4	
Souza-Barbosa et al., 2006 [35]	Brazil	HCTZ	20–25 mg	18	12 w	49.4 ± 7.9	26.4 ± 3.3	7/11	NA	148 ± 11	92 ± 9	Vascular ultrasonography
		Quinapril	20 mg	16	12 w	48.8 ± 8.6	26.3 ± 2.5	7/9	NA	150 ± 14	94 ± 11	
		Irbesartan	150 mg	14	12 w	50.3 ± 7.5	24.5 ± 3.5	6/9	NA	168 ± 15	90 ± 12	
		Quinapril + irbesartan	20 + 150 mg	15	12 w	49.9 ± 5.1	26.6 ± 2.8	6/8	NA	164 ± 17	90 ± 11	
Aznaouridis et al., 2007 [38]	Greece	Captopril	25 mg	25	2 h	57.4 ± 9.6	28.0 ± 4.0	10/15	2 (1–6.5)	148.8 ± 18.9	89.4 ± 9.4	Vascular ultrasonography
		Quinapril	20 mg	25	2 h	56.8 ± 10.4	28.1 ± 4.2	13/12	5 (1–12)	146.6 ± 15.9	92.9 ± 12.2	
		Telmisartan	80 mg	25	2 h	56.3 ± 8.9	28.3 ± 3.9	12/13	2 (1–7)	148.7 ± 19.2	95.9 ± 15.3	
Benndorf et al., 2007 [20]	Germany	Telmisartan	40–80 mg	12	6 w	59.0 ± 7	23.0 ± 3.0	7/5	NA	NA	NA	Vascular ultrasonography
		Nisoldipine	10 mg	13	6 w	56.9 ± 8	27.1 ± 4.1	6/7	NA	NA	NA	
		Telmisartan + Nisoldipine	40 + 10 mg	12	6 w	59.6 ± 8	24.5 ± 4.2	7/5	NA	NA	NA	
Buus et al., 2007 [39]	Denmark	Perindopril	4 mg	15	12 m	49 ± 2	27.1 ± 0.5	10/5	NA	160 ± 4	105 ± 2	Vascular ultrasonography
		Atenolo	50 mg	16	12 m	51 ± 2	26.8 ± 0.6	13/3	NA	158 ± 3	105 ± 1	
Ghiadoni et al., 2007 [15]	Italy	Ramipril	5 mg	21	24 w	NA	NA	NA	NA	163 ± 13	101 ± 6	Vascular ultrasonography
		Ramipril	10 mg	21	24 w	NA	NA	NA	NA	163 ± 13	101 ± 6	
Koh et al., 2007 [40]	Korea	Ramipril	10 mg	34	2 m	46 ± 2	25.24 ± 0.43	NA	NA	155 ± 1	95 ± 1	Vascular ultrasonography
		Candesartan	16 mg	34	2 m		25.22 + 0.43	NA	NA	156 ± 1	95 ± 1	
		Ramipril + Candesartan	10 + 16 mg	34	2 m		25.26 + 0.44	NA	NA	157 ± 1	96 ± 1	
Morimoto et al., 2007 [54]	Japan	Amlodipine	25 mg	25	24 w	58 ± 2	24.2 ± 0.7	12/13	5 ± 1	161 ± 3	96 ± 1	Vascular ultrasonography
		Cilnidipine	10 mg	25	24 w	57 ± 3	24.5 ± 0.9	14/11	6 ± 1	160 ± 3	97 ± 3	
Pasini et al., 2007 [16]	Italy	Zofenopril	15–30 mg	15	8 w	51.5 ± 8.2	25.8 ± 2.2	7/8	NA	152.8 ± 6.7	99.2 ± 4.1	Vascular ultrasonography
		Ramipril	2.5–5 mg	15	8 w	52.2 ± 13.9	24.8 ± 2.6	8/7	NA	152.7 ± 7.6	98.1 ± 4.2	
		Atenolol	50–100 mg	15	8 w	48.6 ± 12.5	25.4 ± 1.4	7/8	NA	150.8 ± 5.5	99.4 ± 3.9	
Hirooka et al., 2008 [55]	Japan	Valsartan	80 mg	9	30 d	57 ± 3	25.7 ± 1.5	2/7	NA	NA	NA	Vascular ultrasonography
		Amlodipine	5 mg	9	30 d	66 ± 3	24.5 ± 1.1	4/5	NA	NA	NA	
Kosch et al., 2008 [30]	Turkey	Valsartan	80 mg	35	12 w	45.4 ± 5	29 ± 5	18/17	31 ± 32 (m)	149.2 ± 13.3	97.5 ± 7	Vascular ultrasonography
		Metoprolol	50 mg	33	12 w	46.2 ± 6	28.2 ± 5	19/14	33 ± 34 (m)	152.9 ± 16.7	98.5 ± 7	
Korkmaz et al., 2008 [31]	Turkey	Quinapril	20 mg	27	4 w	53 ± 9	30 ± 5	13/14	NA	159 ± 14	94 ± 5	Vascular ultrasonography
		Nebivolol	5 mg	27	4 w	52 ± 9	30 ± 4	12/15	NA	163 ± 16	97 ± 8	
Pasini et al., 2008 [17]	Italy	Atenolol	100 mg	20	4 w	55.9 ± 10	26.8 ± 3.3	8/12	NA	152.1 ± 7.9	96.3 ± 4.7	Vascular ultrasonography
		Nebivolol	5 mg	20	4 w				NA			
Yamada et al., 2008 [56]	Japan	Azelnidipine	16 mg	21	8 w	65 ± 9	24.0 ± 2.5	16/5	NA	131 ± 13	79 ± 10	Vascular ultrasonography
		Benidipine	4 mg	21	8 w				NA			
Ghiadoni et al., 2009 [18]	Italy	Perindopril	2–5 mg	31	24 w	48.1 ± 10.7	26.5 ± 2.1	24/7	NA	160 ± 5	100 ± 3	Vascular ultrasonography
		Perindopril	50–100 mg	31	24 w	49.1 ± 9	26.4 ± 2.4	26/5	NA	160 ± 6	101 ± 4	
Jung et al., 2009 [41]	Korea	Telmisartan	80 mg	39	8 w	61 ± 6	24.7 ± 2.4	36/3	NA	153 ± 15	90 ± 13	Vascular ultrasonography
Kim et al., 2009 [42]	Korea	Cilnidipine	10 mg	44	6 m	57.9 ± 9.7	NA	23/21	NA	136.4 ± 11.2	82.2 ± 7.4	Vascular ultrasonography
		Cilnidipine + captopril	10 mg + 25 mg	43	6 m	57.2 ± 10.5	NA	29/14	NA	135.7 ± 12.2	83.6 ± 7.8	
Koh et al., 2009 [43]	Korea	Amlodipine	10 mg	45	8 w	52 ± 2	25.27 ± 0.31	29/16	NA	154 ± 1	95 ± 1	Vascular ultrasonography
Simova et al., 2009 [46]	Bulgaria	Nebivolol	5 mg	14	8 w	45.3 ± 11.5	28 ± 5.2	18/7	NA	152.4 ± 18.5	99.3 ± 9.3	Vascular ultrasonography
		Bisoprolol	5 mg	11	8 w							
Sozen, 2009	Turkey	Irbesartan	300 mg	11	6 w	45 ± 5	27.7 ± 3.9	1/10	NA	NA	NA	Vascular ultrasonography
		Valsartan	160 mg	11	6 w	44 ± 7	27.0 ± 2.9	4/7	NA	NA	NA	
		Fosinopril	10 mg	11	6 w	46 ± 11	26.9 ± 3.6	7/4	NA	NA	NA	
		Quinapril	20 mg	11	6 w	45 ± 8	25.6 ± 3.0	6/5	NA	NA	NA	
Yamanari et al., 2009 [57]	Japanese	Spironolactone	25 mg	14	16 w	77 ± 6	NA	3/11	NA	151 ± 7	79 ± 8	Vascular Ultrasonography
		Chlorthalidone	25 mg	14	16 w	77 ± 6	NA	3/11	NA	149 ± 10	78 ± 7	
Koh et al., 2010 [44]	Korea	Atenolol	100 mg	31	8 w	49 ± 2	24.90 ± 0.41	21/10	NA	156 ± 1	96 ± 1	Vascular ultrasonography
		Amlodipine	10 mg	30	8 w	51 ± 2	25.11 ± 0.36	19/11	NA	155 ± 1	96 ± 1	
		Hydrochlorothiazide	50 mg	31	8 w	48 ± 2	25.30 ± 0.44	20/11	NA	153 ± 1	94 ± 1	
		Ramipril	10 mg	30	8 w	46 ± 1	25.14 ± 0.45	20/10	NA	155 ± 1	94 ± 1	
		Candesartan	16 mg	31	8 w	47 ± 2	25.21 ± 0.43	22/9	NA	156 ± 1	94 ± 1	
Huang et al., 2010 [22]	China	Carvedilol	10 mg	28	6 m	60.2 ± 11.6	NA	18/10	NA	156 ± 7	90 ± 2	Vascular ultrasonography
		Metoprolol	50 mg	29	6 m	62.1 ± 13.8	NA	17/12	NA	158 ± 4	89 ± 3	
Heffernan et al., 2011 [47]	USA	Metoprolol	50 mg	12	4 w	56 ± 2	28 ± 1	NA	NA	138 ± 2	80 ± 2	Vascular ultrasonography
		Atenolol	50 mg	12	4 w					141 ± 2	79 ± 2	
Muiesan et al., 2011 [19]	Italy	Barnidipine	10 mg	20	24 w	50 ± 9.5	26.8 ± 4	15/4	NA	147 ± 12	96 ± 8	Vascular ultrasonography
		HCTZ	12.5 mg	20	24 w	47 ± 9.8	26.4 ± 3	16/4	NA	142 ± 11	93 ± 8	
Takiguchi et al., 2011 [58]	Japan	Olmesartan	20 mg	15	4 w	55 ± 11	25.8 ± 4.2	13/2	NA	152 ± 15	95 ± 10	Vascular ultrasonography
		Amlodipine	5 mg	16	4 w	56 ± 11	24.4 ± 3.1	14/2	NA	149 ± 21	91 ± 14	
Fujimura et al., 2012 [59]	Japan	Eplerenone	NA	20	48 w	NA	24.4 ± 2.6	NA	NA	159.5 ± 14.6	95.3 ± 10.2	Vascular ultrasonography
		Nifedipine	NA	20	48 w	NA	24.2 ± 2.7	NA	NA	159.8 ± 14.4	96.1 ± 10.5	
		Losartan	NA	20	48 w	NA	24.3 ± 2.5	NA	NA	159.7 ± 13.9	95.4 ± 9.8	
Wei et al., 2012 [23]	China	Nisoldipine	10 mg	27	8 w	58.6 ± 7.27	24.8 ± 2.76	41/14	NA	148 ± 9.1	87.8 ± 9.0	Vascular ultrasonography
		Olmesartan	20 mg	28	8 w							
Zepeda et al., 2012 [48]	Chile	Carvedilol	12.5 mg	23		45.6 ± 2.8	27.6 (6.2)	16/7	NA	139 ± 5.1	97.3 ± 6.6	Vascular ultrasonography
		Nebivolol	5 mg	21	12 w	44.9 ± 2.1	26.7 (4.7)	15/6	NA	141 ± 6.3	98.7 ± 5.2	
Sendur et al., 2014 [33]	Turkey	Olmesartan	5 mg	42	8 w	54.9 ± 7.9	30.6 ± 3.8	19/23	NA	154.2 ± 4.0	94.9 ± 2.4	Vascular ultrasonography
		Nebivolol	10 mg	43	8 w	50.1 ± 9.4	30.4 ± 4.7	11/32	NA	151.2 ± 4.1	93.9 ± 2.5	
Takase et al., 2014 [60]	Japan	Losartan + HCTZ	50 mg + 12.5 mg	21	8 w	69.2 ± 7	NA	NA	NA	146 ± 5	78 ± 8	Vascular ultrasonography
		Placebo	NA	21	8 w	69.4 ± 7	NA	NA	NA	148 ± 7	78 ± 9	
Fonseca et al., 2015 [36]	Brazil	Perindopril	4 mg	27	12 w	NA	NA	NA	NA	150 (141NA168)	90 (90NA98)	Vascular ultrasonography
		HCTZ	25 mg	32	12 w	NA	NA	NA	NA	149 (140NA160)	90 (89NA92)	
Gismondi et al., 2015 [37]	Brazil	Benazepril	10 mg	14	12 w	57 (52–62)	29.5 (26.4–34.4)	5/9	NA	147 (128–158)	89 (77–92)	Vascular ultrasonography
		Losartan	50 mg	16	12 w	57 (53–63)	30.3 (27.4–33.2)	6/10	NA	143 (131–153)	81 (78–89)	
Zhao et al., 2017 [24]	China	Amlodipine	5 mg	48	3 m	49.12 ± 12.55	25.01 ± 2.72	22/26	NA	155.2 ± 11.7	95.9 ± 10.2	Vascular ultrasonography
		Amlodipine + atorvastatin	5 mg	52	3 m	48.41 ± 11.16	26.28 ± 3.29	25/27	NA	154.1 ± 10.3	96.3 ± 8.2	
Zhou et al., 2017 [25]	China	Irbesartan	150 mg	46	2 w	63.05 ± 10.13	24.03 ± 2.36	25/21	NA	135 ± 14	78 ± 7	Vascular ultrasonography
		Diltiazem	90 mg	51	2 w	62.32 ± 9.51	23.68 ± 2.71	28/23	NA	136 ± 11	81 ± 10	
		Irbesartan + diltiazem	150 mg + 90 mg	53	2 w	61.82 ± 8.96	24.64 ± 2.40	31/22	NA	139 ± 12	82 ± 10	
Zhang et al., 2020 [26]	China	Placebo	NA	34	30 d	41.2 ± 9.8	24.2 ± 1.34	18/16	NA	151.2 ± 5.5	96.0 ± 1.91	Vascular ultrasonography
		Allisartanisoproxil	240 mg	34	30 d	42.7 ± 9.2	24.0 ± 1.62	17/17	NA	151.1 ± 5.8	95.7 ± 2.2	

**Table 2:** Inconsistency test between direct and indirect treatment comparisons in mixed treatment comparison in FMD.

Side	Direct		Indirect		Difference		*p* > |*z*|
	Coefficient	SE	Coefficient	SE	Coefficient	SE	
Placebo CCB	0.720	1.03	0.710	0.750	0.010	1.275	0.994
Placebo ACEI	8.700	1.580	1.641	0.595	7.059	1.688	0.000
Placebo ARB	0.790	1.053	2.820	0.700	−2.030	1.264	0.108
Placebo diuretic	0.840	1.133	2.081	0.729	−1.241	1.347	0.357
CCB ACEI	1.801	0.619	1.874	0.423	−0.073	0.748	0.922
CCB ARB	1.574	0.398	1.449	0.432	−0.073	0.7475	0.922
CCB *β*-blocker	1.077	0.7567	1.699	0.470	−0.622	0.890	0.485
CCB diuretic	1.039	0.477	0.990	0.474	0.489	0.672	0.942
CCB ARB + CCB	4.900	1.994	1.404	2.191	3.496	2.963	0.238
CCB ACEI + CCB	0.600	1.164	−1.389	173.981	1.989	173.985	0.991
ACEI ARB	0.055	0.476	−0.655	0.426	0.710	0.639	0.267
ACEI *β*-blocker	−0.219	0.533	−0.440	0.549	0.221	0.764	0.773
ACEI diuretic	−1.234	0.881	−1.437	0.520	0.203	1.025	0.843
ACEI CCB + *β*-blocker	−2.5	1.700	−4.990	187.339	2.491	187.352	0.989
ACEI ACEI + ARB	0.605	0.819	−0.213	0.939	0.818	1.246	0.511
ARB *β*-blocker	−0.058	0.641	0.046	0.483	−0.105	0.803	0.896
ARB diuretic	−0.118	0.524	−0.766	0.436	0.647	0.681	0.342
ARB ACEI + ARB	0.580	1.126	0.585	0.784	−0.006	1.373	0.997
ARB ARB + CCB	−0.80	2.172	3.414	2.015	−3.494	2.963	0.238
*β*-Blocker diuretic	−1.170	1.019	−0.364	0.480	−0.806	1.126	0.474
*β*-Blocker ACEI + diuretic	0.400	1.148	−4.524	112.722	4.924	112.729	0.965

SE, standard error; ACEI: angiotensin-converting enzyme inhibitors, ARB: angiotensin receptor blocker, CCB: calcium channel blockers.

**Table 3:** Inconsistency test between direct and indirect treatment comparisons in mixed treatment comparison in BAD.

Side	Direct		Indirect		Difference		*P* > |*z*|
	Coefficient	SE	Coefficient	SE	Coefficient	SE	
CCB ACEI	−0.069	0.151	−0.130	0.122	0.061	0.195	0.753
CCB ARB	−0.286	0.086	−0.128	0.150	−0.158	0.172	0.359
CCB *β*-blocker	−1.69	0.214	−0.211	0.106	0.211	0.239	0.377
ACEI ARB	0.018	0.135	−0.251	0.112	0.269	0.175	0.125
ACEI *β*-blocker	−0.148	0.109	0.070	0.137	−0.218	0.175	0.212
ARB *β*-blocker	0.114	0.103	0.016	0.136	0.097	0.172	0.573

## Data Availability

All data generated or analyzed are included in this article.

## Appendix

Search strategy: we search for all relevant articles published in English until November 1, 2020, in PubMed, Embase, and the Cochrane Library. We conduct the following searches.

PubMed search strategy: ((angiotensin-converting enzyme inhibitors OR captopril OR zofenopril OR enalapril OR ramipril OR quinapril OR perindopril OR lisinopril OR benzazepines OR fosinopril OR alacepril OR cilazapril OR delapril OR imidapril OR moexipril OR rentiapril OR spirapril OR temocapril OR trandolapril) OR (angiotensin receptor antagonists OR azilsartan OR candesartan OR eprosartan OR irbesartan OR losartan OR olmesartan OR tasosartan OR telmisartan OR valsartan) OR (calcium channel blockers OR amlodipine OR aranidipine OR azelnidipine OR barnidipine OR benidipine OR cilnidipine OR clevidipine OR darodipine OR efonidipine OR felodipine OR isradipine OR lacidipine OR manidipine OR lercanidipine OR mepirodipine OR nicardipine OR nifedipine OR niludipin OR nilvadipine OR nimodipine OR nisoldipine OR nitrendipine OR oxodipine OR pranidipine OR ryodipine OR anipamil OR devapamil OR emopamil OR falipamil OR gallopamil OR verapamil OR clentiazem OR diltiazem OR dihydropyridines) OR (adrenergic beta-antagonists OR alprenolol OR bopindolol OR bupranolol OR carteolol OR cloranolol OR mepindolol OR nadolol OR oxprenolol OR penbutolol OR pindolol OR propranolol OR sotalol OR tertatolol OR timolol OR betaxolol OR acebutolol OR bevantolol OR bisoprolol OR epanolol OR celiprolol OR esmolol OR metoprolol OR practolol OR atenolol OR talinolol OR carvedilol OR labetalol OR nebivolol OR butoxamine) OR (acetazolamide OR diuretics OR furosemide OR bumetanide OR torasemide OR ethacrynic acid OR thiazides OR hydrochlorothiazide bendroflumethiazide OR hydroflumethiazide OR chlorothiazide OR polythiazide OR trichlormethiazide OR cyclopenthiazide OR methyclothiazide OR cyclothiazide OR mebutizide OR quinethazone OR clopamide OR chlorthalidone OR mefruside OR clofexamide OR metolazone OR meticrane OR xipamide OR indapamide OR clorexolone OR fenquizone OR amiloride OR triamterene OR benzamil OR spironolactone OR eplerenone OR canrenoate potassium OR canrenone) OR (adrenergic alpha-antagonists OR prazosin OR indoramin OR trimazosin OR doxazosin OR urapidil OR alfuzosin OR silodosin OR tamsulosin OR terazosin) OR (antihypertensive agents)) AND (hypertension OR high blood pressure) AND (flow-mediated vasodilation OR flow mediated dilatation OR nitroglycerine-induced vasodilation OR Vascular Function Tests OR endothelial function)

Cochrane Library search strategy: (1) Angiotensin-Converting Enzyme Inhibitors OR Captopril OR Zofenopril OR Enalapril OR Ramipril OR Quinapril OR Perindopril OR Lisinopril OR Benzazepines OR Fosinopril OR Alacepril OR Cilazapril OR Delapril OR Imidapril OR Moexipril OR Rentiapril OR Spirapril OR Temocapril OR Trandolapril; (2) Angiotensin Receptor Antagonists OR Azilsartan OR Candesartan OR Eprosartan OR Irbesartan OR Losartan OR Olmesartan OR Tasosartan OR Telmisartan OR Valsartan; (3) Calcium Channel Blockers OR Amlodipine OR Aranidipine OR Azelnidipine OR Barnidipine OR Benidipine OR Cilnidipine OR Clevidipine OR Darodipine OR Efonidipine OR Felodipine OR Isradipine OR Lacidipine OR Manidipine OR Lercanidipine OR Mepirodipine OR Nicardipine OR Nifedipine OR Niludipin OR Nilvadipine OR Nimodipine OR Nisoldipine OR Nitrendipine OR Oxodipine OR Pranidipine OR Ryodipine OR Anipamil OR Devapamil OR Emopamil OR Falipamil OR Gallopamil OR Verapamil OR Clentiazem OR Diltiazem OR Dihydropyridines; (4) Adrenergic beta-Antagonists OR Alprenolol OR Bopindolol OR Bupranolol OR Carteolol OR Cloranolol OR Mepindolol OR Nadolol OR Oxprenolol OR Penbutolol OR Pindolol OR Propranolol OR Sotalol OR Tertatolol OR Timolol OR Betaxolol OR Acebutolol OR Bevantolol OR Bisoprolol OR Epanolol OR Celiprolol OR Esmolol OR Metoprolol OR Practolol OR Atenolol OR Talinolol OR Carvedilol OR Labetalol OR Nebivolol OR Butoxamine; (5) Acetazolamide OR Diuretics OR Furosemide OR Bumetanide OR Torasemide OR Ethacrynic Acid OR Thiazides OR Hydrochlorothiazide Bendroflumethiazide OR Hydroflumethiazide OR Chlorothiazide OR Polythiazide OR Trichlormethiazide OR Cyclopenthiazide OR Methyclothiazide OR Cyclothiazide OR Mebutizide OR Quinethazone OR Clopamide OR Chlorthalidone OR Mefruside OR Clofenamide OR Metolazone OR Meticrane OR Xipamide OR Indapamide OR Clorexolone OR Fenquizone OR Amiloride OR Triamterene OR Benzamil OR Spironolactone OR Eplerenone OR Canrenoate Potassium OR Canrenone; (6) Adrenergic alpha-Antagonists OR Prazosin OR Indoramin OR Trimazosin OR Doxazosin OR Urapidil OR Alfuzosin OR Silodosin OR Tamsulosin OR Terazosin; (7) Antihypertensive Agents; (8) (#1 OR #2 OR #3 OR #4 OR #5 OR #6 OR #7); (9) flow-mediated vasodilation OR flow mediated dilatation OR nitroglycerine-induced vasodilation OR Vascular Function Tests OR endothelial function; (10) hypertension OR high blood pressure; (11) (#8 AND #9 AND #10).

Embase search strategy: (1) ‘Angiotensin-Converting Enzyme Inhibitors' OR ‘Captopril' OR ‘Zofenopril' OR ‘Enalapril' OR ‘Ramipril' OR ‘Quinapril' OR ‘Perindopril' OR ‘Lisinopril' OR ‘Benzazepines' OR ‘Fosinopril' OR ‘Alacepril‘ OR ‘Cilazapril' OR ‘Delapril' OR ‘Imidapril' OR ‘Moexipril' OR ‘Rentiapril' OR ‘Spirapril' OR ‘Temocapril' OR ‘Trandolapril'; (2) ‘Angiotensin Receptor Antagonists' OR ‘Azilsartan' OR ‘Candesartan' OR ‘Eprosartan' OR ‘Irbesartan' OR ‘Losartan' OR ‘Olmesartan' OR ‘Tasosartan' OR ‘Telmisartan' OR ‘Valsartan'; (3) ‘Calcium Channel Blockers' OR ‘Amlodipine' OR ‘Aranidipine' OR ‘Azelnidipine' OR ‘Barnidipine' OR ‘Benidipine' OR ‘Cilnidipine' OR ‘Clevidipine' OR ‘Darodipine' OR ‘Efonidipine' OR ‘Felodipine' OR ‘Isradipine' OR ‘Lacidipine' OR ‘Manidipine' OR ‘Lercanidipine' OR ‘Mepirodipine' OR ‘Nicardipine' OR ‘Nifedipine' OR ‘Niludipin' OR ‘Nilvadipine' OR ‘Nimodipine' OR ‘Nisoldipine' OR ‘Nitrendipine' OR ‘Oxodipine' OR ‘Pranidipine' OR ‘Ryodipine' OR ‘Anipamil' OR ‘Devapamil' OR ‘Emopamil' OR ‘Falipamil' OR ‘Gallopamil' OR ‘Verapamil' OR ‘Clentiazem' OR ‘Diltiazem' OR ‘Dihydropyridines'; (4) ‘Adrenergic beta-Antagonists' OR ‘Alprenolol' OR ‘Bopindolol' OR ‘Bupranolol' OR ‘Carteolol' OR ‘Cloranolol' OR ‘Mepindolo'l OR ‘Nadolol' OR ‘Oxprenolol' OR ‘Penbutolol' OR ‘Pindolol' OR ‘Propranolol' OR ‘Sotalol' OR ‘Tertatolol' OR ‘Timolol' OR ‘Betaxolol' OR ‘Acebutolol' OR ‘Bevantolol' OR ‘Bisoprolol' OR ‘Epanolol' OR ‘Celiprolol' OR ‘Esmolol' OR ‘Metoprolol' OR ‘Practolol' OR ‘Atenolol' OR ‘Talinolol' OR ‘Carvedilol' OR ‘Labetalol' OR ‘Nebivolol' OR ‘Butoxamine'; (5) ‘Acetazolamide' OR ‘Diuretics' OR ‘Furosemide' OR ‘Bumetanide' OR ‘Torasemide' OR ‘Ethacrynic Acid' OR ‘Thiazides' OR ‘Hydrochlorothiazide Bendroflumethiazide' OR ‘Hydroflumethiazide' OR ‘Chlorothiazide' OR ‘Polythiazide' OR ‘Trichlormethiazide' OR ‘Cyclopenthiazide' OR ‘Methyclothiazide' OR ‘Cyclothiazide' OR ‘Mebutizide' OR ‘Quinethazone' OR ‘Clopamide' OR Chlorthalidone OR ‘Mefruside' OR ‘Clofenamide' OR ‘Metolazone' OR Meticrane OR ‘Xipamide' OR ‘Indapamide' OR Clorexolone OR ‘Fenquizone' OR ‘Amiloride' OR ‘Triamterene' OR ‘Benzamil' OR ‘Spironolacton'e OR ‘Eplerenone' OR ‘Canrenoate Potassium' OR ‘Canrenone'; (6) ‘Adrenergic alpha-Antagonists' OR ‘Prazosin' OR ‘Indoramin' OR ‘Trimazosin' OR ‘Doxazosin' OR ‘Urapidil' OR ‘Alfuzosin' OR ‘Silodosin' OR ‘Tamsulosin' OR ‘Terazosin'; (7) ‘Antihypertensive Agents'; (8) (1 OR 2 OR 3 OR 4 OR 5 OR 6 OR 7); (9) ‘flow-mediated vasodilation' OR ‘flow mediated dilatation' OR ‘nitroglycerine-induced vasodilation' OR ‘Vascular Function Tests' OR ‘endothelial function'; (10) ‘hypertension' OR ‘high blood pressure'; (11) (8 AND 9 AND 10). https://downloads.hindawi.com/journals/ijhy/2022/2432567.f1.docx
